# Improving the cytotoxic response of tumor-infiltrating lymphocytes towards advanced stage ovarian cancer with an oncolytic adenovirus expressing a human vIL-2 cytokine

**DOI:** 10.1038/s41417-023-00658-3

**Published:** 2023-09-04

**Authors:** D. C. A. Quixabeira, E. Jirovec, S. Pakola, R. Havunen, S. Basnet, J. M. Santos, T. V. Kudling, J. H. A. Clubb, L. Haybout, V. Arias, S. Grönberg-Vähä-Koskela, V. Cervera-Carrascon, A. Pasanen, M. Anttila, J. Tapper, A. Kanerva, A. Hemminki

**Affiliations:** 1 Cancer Gene Therapy Group, Translational Immunology Research Program, Faculty of Medicine, University of Helsinki, Helsinki, Finland; 2 TILT Biotherapeutics Ltd, Helsinki, Finland; 3 Helsinki University Hospital Comprehensive Cancer Center, Helsinki, Finland; 4 Department of Pathology, University of Helsinki and Helsinki University Hospital, Helsinki, Finland; 5 Pathology, Finnish Food Authority, Helsinki, Finland; 6 Department of Obstetrics and Gynecology, Helsinki University Central Hospital, Helsinki, Finland

**Keywords:** Drug development, Cancer immunotherapy

## Abstract

While the presence of tumor-infiltrating lymphocytes (TILs) associates with improved survival prognosis in ovarian cancer (OvCa) patients, TIL therapy benefit is limited. Here, we evaluated an oncolytic adenovirus coding for a human variant IL-2 (vIL-2) cytokine, Ad5/3-E2F-d24-vIL2 (vIL-2 virus), also known as TILT-452, as an immunotherapeutic strategy to enhance TIL responsiveness towards advanced stage OvCa tumors. Fragments of resected human OvCa tumors were processed into single-cell suspensions, and autologous TILs were expanded from said samples. OvCa tumor specimens were co-cultured with TILs plus vIL-2 virus, and cell killing was assessed in real time through cell impedance measurement. Combination therapy was further evaluated in vivo through a patient-derived xenograft (PDX) ovarian cancer murine model. The combination of vIL-2 virus plus TILs had best cancer cell killing ex vivo compared to TILs monotherapy. These results were supported by an in vivo experiment, where the best OvCa tumor control was obtained when vIL-2 virus was added to TIL therapy. Furthermore, the proposed therapy induced a highly cytotoxic phenotype demonstrated by increased granzyme B intensity in NK cells, CD4+ T, and CD8+ T cells in treated tumors. Our results demonstrate that Ad5/3-E2F-d24-vIL2 therapy consistently improved TILs therapy cytotoxicity in treated human OvCa tumors.

## Introduction

Adoptive cell transfer with autologous tumor-infiltrating lymphocytes (TILs) has been proposed as an immunotherapy for the treatment of certain immune-infiltrated solid tumors in advanced stage or metastatic lesions [1–3]. Trial results with up to 50% objective response and up to 20% durable response in advanced melanoma patients [4, 5] have prompted interest to investigate other potential tumor targets for adoptive TIL cell therapy [6, 7]. In this context, ovarian cancer (OvCa) appears as a prospective candidate given the natural occurrence of intratumoral lymphocytes and demonstrated presence of specific anti-tumor reactive TILs in OvCa lesions [8, 9]. Moreover, TILs therapy could potentially provide an alternative therapeutic option for advanced stage patients, who have a high recurrence rate and a poor survival prognosis [10].

Clinical trials employing autologous TIL adoptive transfer for the treatment of OvCa have progressed with optimization of TILs protocols yielding higher cell amounts after expansion, and implementation of pre-conditioning chemotherapy for improved clinical response [7, 9, 11–13]. However, clinical benefits are sporadic and short-term stable disease seems to be the most frequent outcome [14]. Comprehensive studies of OvCa tumors´ milieu suggest limited efficacy of naturally occurring TILs to mount an anti-tumor response, and a hostile OvCa tumor microenvironment (TME) as relevant contributing factors for therapy failure [13, 15–17].

OvCa is the third most common gynecological cancer type with a poor 5-year survival rate, with the majority of patients being diagnosed at a late stage of the disease [18]. Treatment options for OvCa involve debulking surgery and chemotherapy options with the combination approach of carboplatin and paclitaxel [19]. More recently, immune checkpoint inhibitors have also been used for the treatment of OvCa, however, with no much therapeutic benefit observed [19].

Besides the presence of malignant transformed cancer cells and intratumoral lymphocytes, immune cells of suppressive nature, such as T regulatory (TReg) cells, tumor associated macrophages (TAM), and myeloid derived suppressive cells (MDSC) are enriched in OvCa tumors [20]. Presently, circumventing the OvCa immunosuppressive niche represents a challenge for the success of adoptive cell immunotherapy strategies, including adoptive transfer of autologous TILs [21–23]. Therefore, a therapy with potential to reshape an immunosuppressive TME towards a pro-inflammatory state, seems to be a compelling alternative to enable TILs therapy response in OvCa tumors.

Oncolytic viruses (OV) constitute an attractive class of immunotherapeutic agents for the treatment of human solid tumors [24]. Selective cell infection and cell lysis are some of the OV characteristics that stimulate a potent immune response through release of damage-associated and pathogen-associated molecular patterns and tumor-associated antigens in the TME [25, 26]. Furthermore, OVs can be modified to insertion of human immunomodulatory transgenes. Particularly, oncolytic adenoviruses stand out as a promising family of OV immunotherapeutic candidates as notable immunogenic agents with demonstrated potential to engage the generation of de novo tumor immunity through facilitation of tumor epitope spreading [25].

In the present study, we propose the use of an engineered oncolytic adenovirus coding for a variant interleukin-2 (vIL-2) cytokine, Ad5/3-E2F-d24-vIL2 (vIL-2 virus), as a combination immunotherapy to enhance the cytotoxic potential of TILs in advanced stage human OvCa tumors. The vIL-2 virus features a virus backbone structure, Ad5/3-E2F-d24, with demonstrated selective cancer cell replication and lysis [27]. Moreover, the Ad5/3-E2F-d24-vIL2 virus serves as an expression vector for the vIL-2 cytokine transgene in the treated tumor site [28].

The virus expression of a vIL-2 cytokine intends to stimulate effector lymphocytes, such as natural killer (NK), CD4+ T, and CD8+ T cells over TReg cells in treated tumors [28]. Modifications made on the vIL-2 cytokine binding site to the IL-2 receptor (IL-2R) [29] results in enhanced engagement affinity of vIL-2 cytokine to the subunit IL-2Rβ (CD122) and moderately to IL-2Rγ (CD132) receptor subunit. While binding to IL-2Rα (CD25) domain to the IL-2R is not needed for effector lymphocytes to exert biological functions [30]. This represents a therapeutic advantage in tumors distinctly affected by TReg mediated-immunosuppression, because TReg cells rely on the integral IL-2Rαβγ trimeric receptor engagement for cell functionality [31]. Moreover, vIL-2 cytokine is a good therapeutic asset to minimize wild-type IL-2 systemic toxicity effects frequently reported in treated patients, such as capillary leak syndrome and neurotoxicity [32].

Previously, we demonstrated that vIL-2 virus improved tumor control and survival as a monotherapy in an in vivo hamster pancreatic cancer model, compared to its wild-type IL-2 virus counterpart [28]. Here, the Ad5/3-E2F-d24-vIL2 virus (aka TILT-452) is proposed as an enabler for TIL therapy for the treatment of advanced stage OvCa human tumors.

## Methods

### Surgical patient samples

Fragments of surgically resected human ovarian cancer (OvCa) tumors were received from the Helsinki University Hospital (HUS). Specimens were processed into single-cell suspension upon arrival following a human cancer sample processing protocol [33]. In total, twelve samples with confirmed OvCa diagnosis were included in the present study. Patient characteristics and diagnosis stage overview can be found in Table 1.

**Table 1:** Patient characteristics and diagnosis information.

Patient ID	Age	Diagnosis	Specimen resection site	Primary tumor location	Prior cancer treatments
HUSOV1	57	HGSC Stage IIIB	Ovary	Fallopian tube	–
HUSOV2	76	HGSC Stage IVB	Greater omentum	Fallopian tube	–
HUSOV3	75	HGSC Stage IVB	Greater omentum	Fallopian tube	Carboplatin+ paclitaxel
HUSOV4	79	HGSC Stage IVB	Greater omentum	Fallopian tube	–
HUSOV5	76	HGSC Stage IVB	Greater omentum	Fallopian tube	–
HUSOV6	35	LGSC Stage IVB	Greater omentum	Ovary	–
HUSOV9	50	HGMEC of ovary (clear cell and endometrioid). Stage IC2	Ovary	Left ovary	–
HUSOV10	65	HGSC Stage IVB	Greater omentum	Left ovary	Carboplatin+ paclitaxel
HUSOV12	73	HGSC Stage IVB	Greater omentum	Fallopian tube	Carboplatin
HUSOV13	66	HGSC Stage IVB	Greater omentum	Ovary	–
HUSOV15	79	HGSC Stage IVB	Ovary	Right ovary	Carboplatin
HUSOV16	62	HGSC Stage IVB	Greater omentum	Fallopian tube	Carboplatin+ paclitaxel

Partial data was published as Quixabeira et al., 2022 at ESMO Immuno-Oncology 2022.

*HGSC* high grade serous carcinoma, *LGSC* low grade serous carcinoma.

### Oncolytic adenoviruses

The construction of genetically engineered Ad5/3-E2F-d24 virus, also known as virus backbone, and Ad5/3-E2F-d24-vIL2 (vIL-2 virus) have been previously described [27, 28].

### Expansion of autologous TILs from ovarian cancer tumors

The generation of TILs used in the ex vivo adoptive cell therapy co-cultures and in the in vivo animal experiment followed the adapted “young” TILs expansion protocol [12]. Briefly, ovarian cancer surgical specimens were cut into smaller fragments and put into culture in six-well G-Rex plates (Wilson Wolf Manufacturing, MN, USA) with TILs media [12, 33]. After seven days, the culture content was passed through 70 µm and 40 µm strainers, and the cells were pelleted and stored at −140 °C. For experimental use, TILs were further expanded according to a modified TILs rapid expansion protocol [12]. Initially, day-seven TILs were rested in TILs media for 24 h. Then, TILs were co-cultured with allogeneic irradiated (40 Gy) PBMCs in a ratio of 1–200, and supplemented with TILs media, rapid expansion media, and 30 ng/ml of anti-CD3 (clone OKT3, Thermo Fisher Scientific). Plates were kept in culture at 37 °C for a total of 14 days, and TILs concentration was adjusted to 5 × 10^6^ cells/cm^2^ whenever needed.

### Ovarian cancer cell killing assessment with real-time cell impedance system

The ability of Ad5/3-E2F-d24-vIL2 virus to enhance TILs cell killing potential was assessed ex vivo in OvCa co-cultures through a cell impedance measurement. OvCa tumor digests were plated in duplicates at 5 × 10^4^ cells/well into pre-coated impedance plates (E-Plate 16, 300601150, Agilent, CA, USA) with 5 µg/ml of human fibronectin (ECM001, Sigma Aldrich, MI, USA), and incubated for 24 h. Prior to co-culture, day 14 expanded TILs were rested in TILs media for 24 h. TILs were then harvested and added to the OvCa cultures in a 3:1 effector-to-target (E:T) ratio. Concomitantly, co-cultures were treated either with 100 vp/cell of Ad5/3-E2F-d24 or Ad5/3-E2F-d24-vIL2 virus. Appropriate controls were used accordingly. Impedance plates were scanned every 15 min for a total of 190 hours with the impedance system xCELLigence Real-Time Cell Analysis (RTCA) DP instrument (Agilent, CA, USA). Final data were presented as normalized cell index.

### Immune studies of OvCa co-cultures

In addition to cell killing experiments, flow cytometry was performed in the OvCa co-cultures based on sample availability. OvCa sample digests (3.5 × 10^5^ cells/well) were co-cultured with their respective autologous TILs in 3:1 (E:T) ratio and further treated with 100 vp/cell of Ad5/3-E2F-d24 or Ad5/3-E2F-d24-vIL2 virus. Each condition was plated in triplicate and appropriate controls were applied. Co-cultures were kept in culture for 6 days, when cells were harvested and stained with fluorochrome-conjugated antibodies for flow cytometry analyses. Intracellular staining was performed with BD GolgiPlug™ containing Brefeldin A (555028, BD, NJ, USA) and cell permeabilization with BD Cytofix/Cytoperm™ Plus Fixation/ Permeabilization Kit (555028, BD, NJ, USA), performed according to manufacturer protocols. Cell fluorescence was acquired with NovoCyte Quanteon Flow Cytometer Systems (Agilent, CA, USA), upon acquisition of 90,000 to 100,000 events per well. Cells gating and data processing were done with FlowJo v.10.6.1 (FlowJo LLC, OR, USA). The list of all antibodies used can be found in Supplementary Table 1.

### Animal experiment with an OvCa PDX tumor model

The generation of the OvCa cell line used in the patient-derived xenograft (PDX) in vivo model has been previously described [34]. Patient-derived OvCa cells were injected (3.5 × 10^6^ cells/animal) in the lower left back of 27 immunodeficient female, 5–10 weeks old, NOD.Cg-PrkdcscidIl2rgtm1Sug/JicTac mice (Taconic Biosciences GmbH, Leverkusen, DE). PBMCs from the same patient were used for the humanization of the mice used in the experiment. In addition, to assure sufficient cell number for the humanization of all animals included in the in vivo study, patient-derived PBMCs were expanded using an adapted “young” TILs protocol [12, 34]. Prior animal injection, expanded PBMCs were rested in TILs media for 24 h.

When ovarian cancer PDX tumors reached ~5–6 mm in the longest diameter, animals were randomized into one of the experimental groups: 7 animals per group in mock, TILs monotherapy, TILs plus Ad5/3-E2F-d24 groups, and 6 animals in the combination of TILs plus vIL-2 virus. Expanded PBMCs (5.0 × 10^6^ cells/animal) were given intraperitoneally (i.p.). Subsequently, on day 0, injections of Ad5/3-E2F-d24 or Ad5/3-E2F-d24-vIL2 virus (1×10^9^ vp/animal) were given intratumorally (i.t.), and repeated on days 3, 6, and 9. On day 1, autologous TILs (8.5×10^6^ cells/animal) injections were administrated once i.p. to all cell therapy-treated animals [35]. Tumor progression was measured every two days with a digital caliper, and volumes calculated with the formula (length × width^2^)/2. Tumor growth percentage was calculated by normalizing each measurement to their respective day 0 volumes. On day 12, animals were euthanized and blood, tumors and selected organs (heart, lung, kidney, liver, and spleen) collected for further studies. Of note, the investigators were not blinded to the group allocation during the treatments.

For blood sample processing, erythrocytes were initially lysed with ACK buffer, and white blood cells stored at −80 °C. Collected OvCa tumors were mechanically disrupted into single-cell suspension, and cells frozen down at −80 °C. Processed blood and tumor samples were stained for flow cytometry cell surface markers according to the manufacturer´s instructions. In addition, tumors were further stained for intracellular staining as described above, and transcription factor stained according to the True-Nuclear™ Transcription Factor Buffer Set (424401, BD, NJ, USA) protocol. Flow cytometry runs were done following the same parameters as described in the previous section. The antibodies list can be found in Supplementary Table 1.

### Human ovarian cancer tumors baseline immunohistochemistry and mouse healthy organs toxicity studies

Immune studies of infiltrating lymphocyte subsets at the OvCa tumor specimens at baseline were done as previously described [33]. Slides were then stained for hematoxylin eosin (HE) and with antibodies staining for CD4, CD8, CD56, and PD-L1 (Supplementary Table 2) expressing cells for the immunohistochemistry (IHC) analysis. Results were analysed by an experienced pathologist, who applied a commonly used clinical semi-quantitative scoring system for tumor-infiltrating lymphocytes distribution in tumors. Digital scans of slides were taken using 3DHISTECH Pannoramic 250 FLASH II digital slide scanner.

In addition, HE slides were confectioned from liver, heart, kidney, lung, and spleens of day 12 euthanized mice, and histopathological evaluation of therapy toxicity studied. A veterinary pathologist interpreted the results in a blind manner.

### Statistical analysis

Flow cytometry data and ex vivo co-culture results were interpreted by unpaired *t*-test with or without Welch’s correction. The normality of the tumor growth data was evaluated with Shapiro-Wilk test. Two-way ANOVA with post-hoc Tukey correction was applied to assess treatment differences on tumor response on day 12. Graphical representation of the data, as well as statistical tests analyses were done with GraphPad Prism v.8.4.2, (GraphPad Software Inc, CA, USA).

## Results

### A set of ovarian cancer patient tumors shows marked presence of TILs at baseline

**Fig. 1:**
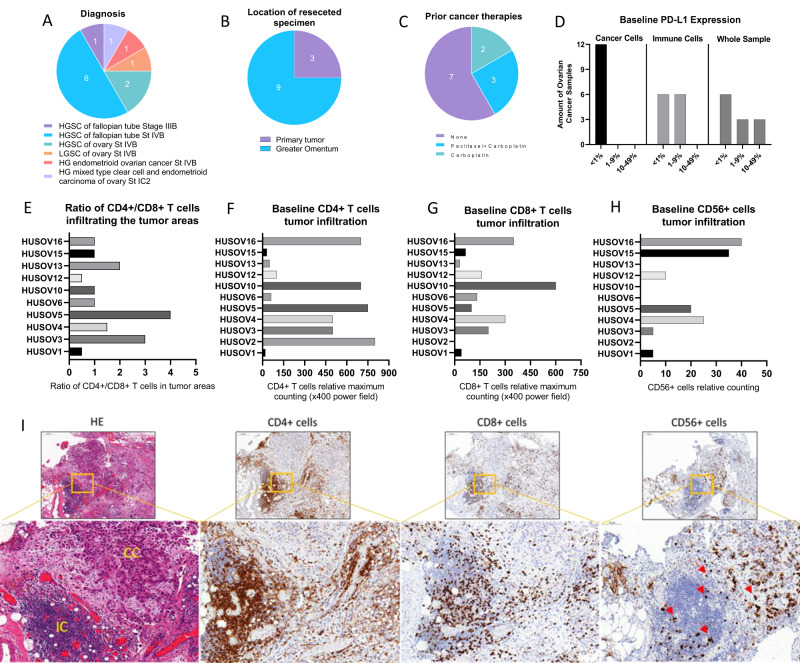
Evaluation of baseline immune-status by immunohistochemistry and diagnosis characteristics in patient-derived ovarian cancer samples. Upon arrival, fragments of ovarian cancer samples were fixed and embedded into paraffin blocks, and staining with HE and immunohistochemistry for CD4+ T, CD8+ T, CD56+, and PD-L1+ cells were performed. **A**–**C** Chart graphs detailing ovarian cancer characteristics on (**A**) diagnosis, (**B**) location of resected specimen, and (**C**) prior cancer therapies. **D** Expression of PD-L1 percentages levels on cancer cells, immune cells and overall counting in ovarian cancer samples. **E** Ratio of CD4+/CD8+ T cell infiltration across study samples. **F** Baseline maximum counting of CD4+ T cell infiltration across all ovarian cancer tumors in ×400 power field. **G** Baseline maximum counting of CD8+ T cell infiltration across all ovarian cancer tumors in ×400 power field. **H** Baseline relative counting of CD56+ infiltrating lymphocyte present in each ovarian cancer samples. **I** Photos of slides representing lymphocyte infiltration in an ovarian cancer samples. From left to right, HE staining showing in yellow cancer cells (CC) and immune cells (IC) grouping, CD4+ T cells (brown), CD8+ T cells (brown), and CD56+ cells (red arrows) distribution in the same tumor area. IHC photos from HUSOV16 slides were used to exemplify the lymphocyte infiltration pattern. Upper row ×26 magnification (scale bar 200 µm) and lower row ×33 magnification (scale bar 100 µm). Partial data was published as Quixabeira et al, 2022 at ESMO Immuno-Oncology 2022.

Twelve specimens of OvCa cancer presenting a diverse range of histological OvCa subtypes were included in the present study (Fig. 1A). Metastatic lesions in the greater omentum represented 9 out of 12 specimens collected, while only three were resected from primary tumors (Fig. 1B). Regarding prior cancer therapies, 7 patients were naïve to treatments, 5 patients had received neo-adjuvant chemotherapy being 3 patients with carboplatin plus paclitaxel and 2 patients with carboplatin only (Fig. 1C). Immunohistochemistry (IHC) analysis of programmed death-ligand 1 (PD-L1) expression at baseline demonstrated that all 12 samples presented <1% of PD-L1 expression in cancer cells, while PD-L1 in immune cells varied from <1% in half of the samples, and up to 9% in the other half (Fig. 1D). Evaluation of IHC staining for TILs suggest that OvCa samples are predominantly infiltrated by CD4+ T cell compared to CD8+ T cell in a CD4+/CD8+ T cell ratio analysis (Fig. 1E). Infiltration of CD4+ T (Fig. 1F) was observed across all OvCa samples, CD8+ T cell were found in 10 out of 11 samples (Fig. 1G), while CD56+ cell were found in 7 out of 11 samples, and in lower amounts compared to CD4+ and CD8+ T cell (Fig. 1H). Example of TIL baseline findings represented by HUSOV16 sample (Fig. 1I).

### Ad5/3-E2F-d24-vIL2 virus promotes consistent cancer cell killing of ovarian cancer samples treated with autologous TILs ex vivo

**Fig. 2:**
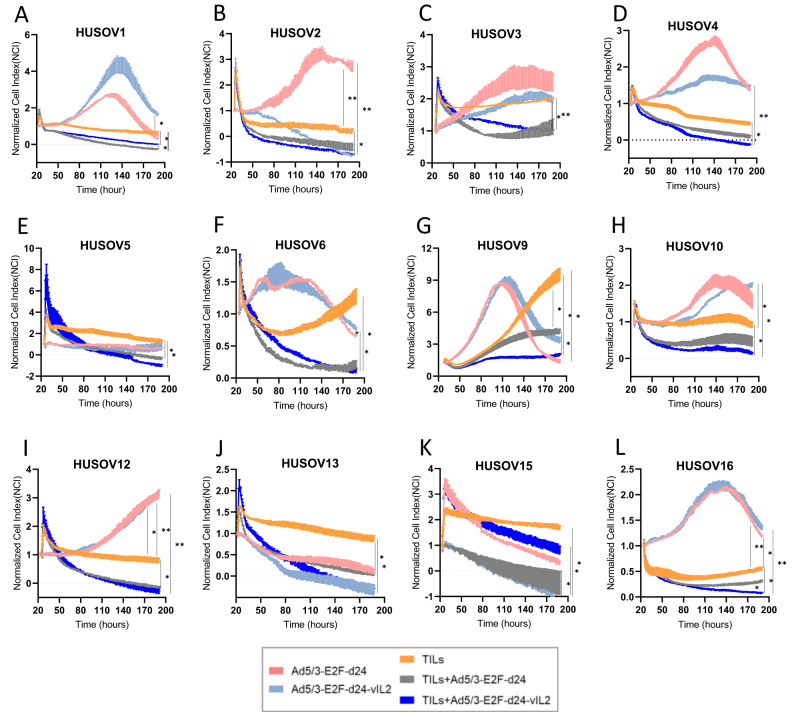
Cancer cell killing study of ovarian cancer tumor digests treated with Ad5/3-E2F-d24-vIL2 and TIL therapy. **A**–**L** Graphs representing individual cell impedance measurements in real time of ovarian cancer tumor samples in response to treatment with Ad5/3-E2F-d24-vIL2 virus, TIL therapy, and Ad5/3-E2F-d24-vIL2 virus in combination with TIL therapy. Control groups with the Ad5/3-E2F-d24 virus were also applied. Tumor single cell suspensions were plated (5 × 10^4^ cells/well) in duplicates in pre-coated plates with human fibronectin (5 μg/ml). After 24 h of incubation, samples were treated with day 14 expanded autologous TILs (3:1) effector to target ratio (E:T) concomitantly to 100vp/cell of Ad5/3-E2F-d24 or Ad5/3-E2F-d24-vIL2 virus. Plates were scanned every 15 min for a total of 190 h. Monotherapy groups respective to each therapy were used as controls of the therapy response. Data sets are presented as normalized cell index over the period of observation and analysed for statistical significance by unpaired *T* test and presented as mean +− SEM. Data is *p < 0.05, **p < 0.01.

To evaluate the cancer cell killing capacity of the proposed combination therapy, OvCa tumor digests were co-cultured with autologous TILs and vIL-2 virus, and therapy response observed in real-time (Fig. 2A–L). Comparable efficacy between virus monotherapies and TILs therapy plus viruses was achieved only in HUSOV9 for virus backbone, and in HUSOV2, HUSOV13, and HUSOV15 for vIL-2 virus treated samples (Fig. 2B, G, J, K). When directly analysing the results of autologous TILs monotherapy versus backbone virus plus TILs, the latter performed better in 7 out of 12 samples, with statistical significance (p < 0.05) (HUSOV1, HUSOV3, HUSOV5, HUSOV6, HUSOV9, HUSOV12, and HUSOV13) (Fig. 2A, C, E–G, I–J). Interestingly, consistent cancer cell killing response in all samples except HUSOV15 was achieved only when vIL-2 virus was added to the autologous TIL therapy in OvCa co-cultures compared to the TILs alone group (*p* < 0.05) (Fig. 2A–L). The vIL-2 virus plus TILs combination also demonstrated improved cell killing compared to the TILs plus unarmed virus in 7 out of 12 OvCa tumor digests (HUSOV2, HUSOV4, HUSOV5, HUSOV9, HUSOV10, HUSOV13, and HUSOV16), with statistically significant results (*p* < 0.05) in HUSOV9 and HUSOV16 (Fig. 2B, D, E, G, H, J, L).

### Effector lymphocyte cytotoxicity is enhanced in ovarian cancer co-cultures treated with vIL-2 virus in combination with TILs therapy

**Fig. 3:**
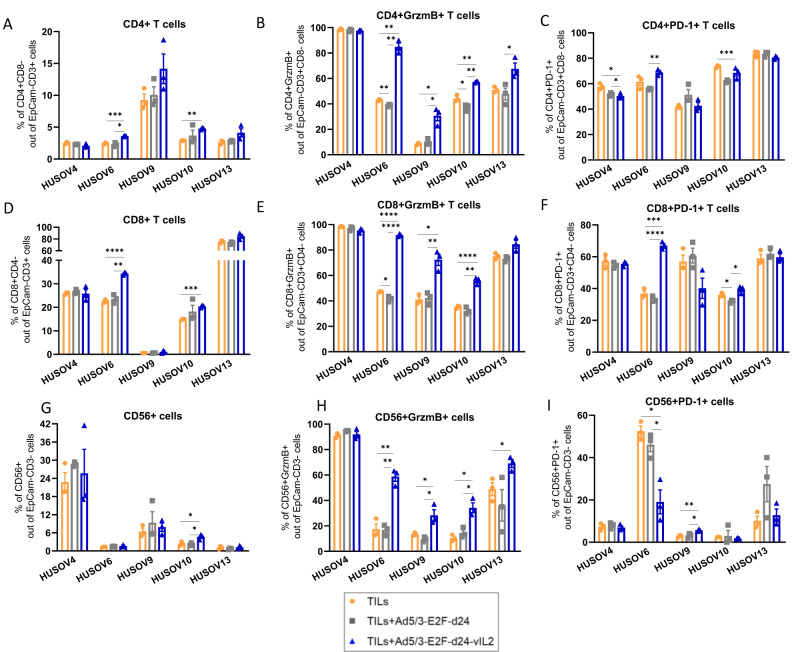
Ovarian cancer co-cultures immune study of effector lymphocytes response to the combination treatment. 3.5 × 10^5^ cells of HUSOV4, HUSOV6, HUSOV9, HUSOV10, and HUSOV13 ovarian cancer tumor digests were plated and incubated for 24 h. Then, samples were treated with their respective day 14 expanded autologous TILs at (3:1) effector to target ratio. In addition, 100vp/cell of either Ad5/3-E2F-d24 virus or Ad5/3-E2F-d24-vIL2 were added to the co-cultures. Autologous TILs treatment as a monotherapy was used as a control of the assay. Co-cultures were incubated at 37 °C and cells were harvested for flow cytometry analyses on day 6. Percentage of (**A**) CD4+ T cells, (**B**) granzyme B CD4+ T cells, and (**C**) PD-1 + CD4+ T cells in day 6 co-cultures. **D** Levels of CD8+ T cells, **E** granzyme B CD8+ T cells, and (**F**) PD-1 + CD8+ T cells in the treated ovarian cancer digests. Percentage of (**G**) CD56+ cells, (**H**) granzyme B CD56+ cells, and (**I**) PD-1 + CD56+ cells in day 6 treated co-cultures. Data sets were analysed for statistical significance by unpaired *T* test with Welch’s correction and presented as mean +− SEM. *p < 0.05, **p < 0.01, ***p < 0.001, ****p < 0.0001.

In addition to cell killing assays of tumor digests, we further studied the immune response of effector lymphocytes in the OvCa co-cultures to the treatment with vIL-2 virus in conjunction with TILs therapy. OvCa samples evaluated in the immune studies were included based on the criteria of specimens’ availability to carry out the assays with inclusion of technical triplicates and appropriate controls for each condition studied. Analyses performed on cells collected on day 6 demonstrate a statistically significant increase in overall CD4+ T cell population in the vIL-2 virus plus TILs group, compared to the TILs monotherapy or virus backbone plus TILs group in HUSOV6 co-culture (*p* < 0.05) (Fig. 3A). In HUSOV10, vIL-2 virus plus TILs also showed higher CD4+ T cell frequencies compared to TILs monotherapy, while no changes were detected in the other samples studied.

A different scenario was observed when the percentage of cytotoxic CD4+ T cells was analysed. Treatment with vIL-2 virus plus TILs stimulated statistically significant higher proportions (*p* < 0.05) of CD4+ T cells expressing granzyme B (GrzmB) compared to the TILs monotherapy (HUSOV6, HUSOV9, and HUSOV10) and backbone virus plus TILs (HUSOV6, HUSOV9, HUSOV10, and HUSOV13) (Fig. 3B). Statistically significant (*p* < 0.05) lower percentage of CD4+ T cells expressing programmed cell death protein 1 (PD-1) in co-cultures after vIL-2 virus plus TILs treatment in HUSOV4 compared to TILs monotherapy and to TILs in combination with virus backbone (Fig. 3C). On the contrary, CD4 + PD-1 + T cells were found in higher percentages in vIL-2 virus plus TILs compared to its unarmed combination group (*p* < 0.01) and relative to TILs monotherapy (*p* < 0.001), in HUSOV6 and HUSOV10 respectively.

Regarding the CD8+ T cell subset, the vIL-2 virus plus TILs treatment induced a statistically significant (*p* < 0.01) increase in frequencies of CD8+ T cells in treated HUSOV6 and HUSOV10 co-cultures in comparison to other treatment groups, and TILs monotherapy, respectively (Fig. 3D). Noticeably, combination therapy of vIL-2 virus plus TILs induced statistically significant higher frequencies (*p* < 0.05) of cytotoxic CD8+ T cells in HUSOV6, HUSOV9, and HUSOV10 treated co-cultures relative to the other experimental groups (Fig. 3E). Also, a trend towards upregulation of these cells in HUSOV13 sample was observed in the proposed therapy, although with no statistical significance. Expression of PD-1 by CD8+ T cells was found to be augmented with statistical significance (*p* < 0.001) only in HUSOV6 and in HUSOV10 co-cultures treated with vIL-2 virus plus TILs therapy relative to the other treatment groups and the TILs plus virus backbone, respectively (Fig. 3F). In HUSOV10, TILs monotherapy induced higher proportions of CD8 + PD-1 + T cells compared to TILs plus virus backbone treatment (*p* < 0.05).

In addition, changes in CD56+ cell (mainly NK cells) percentages and cytotoxicity status were assessed in treated OvCa tumor digests. Presence of NK cells were detected across all treated co-cultures with a higher percentage found in HUSOV4, compared to the other samples, where CD56+ cells were initially detected at the baseline of the tumor fragments collected (Fig. 3G). Of note, the proposed combination immunotherapy of vIL-2 virus with autologous TILs was the only treatment to upregulate cytotoxic CD56+ expressing GrzmB cells with statistical significance (*p* < 0.05) compared to the other treatment strategies tested (Fig. 3H). The only exception was observed in HUSOV4, where percentage of cytotoxic NK cells did not vary regardless of the treatment used. Presence of CD56 + PD-1+ cells was increased in TILs monotherapy and in virus backbone plus TILs groups compared to vIL-2 virus plus TILs with statistical significance (*p* < 0.05) in HUSOV6 (Fig. 3I). While in HUSOV9, the vIL-2 virus plus TILs treatment showed statistically significant higher proportions of those cells compared to the other experimental groups (*p* < 0.05).

### TILs therapy delivers best ovarian cancer anti-tumor response in vivo when used in conjunction with vIL-2 virus therapy

**Fig. 4:**
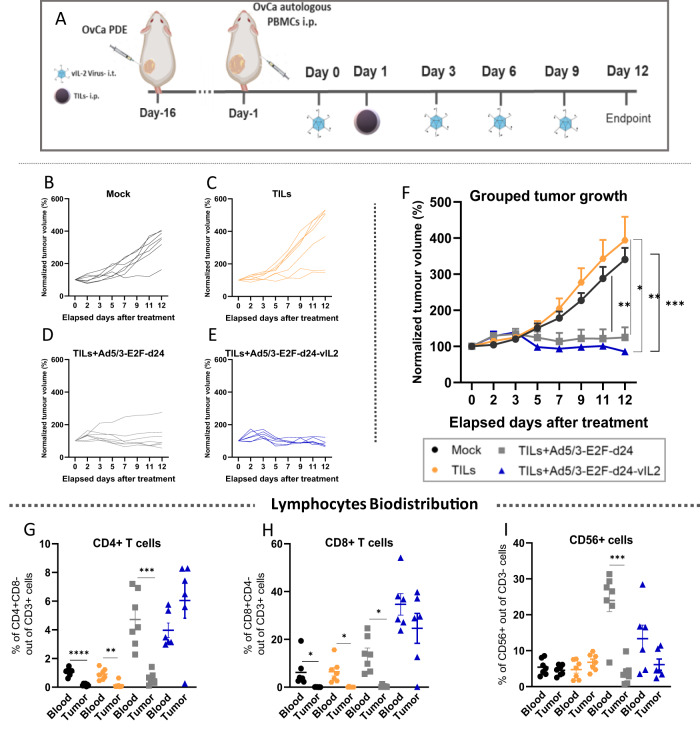
Evaluation of vIL-2 virus plus TILs therapy efficacy in an ovarian cancer PDX tumor model. Patient-derived ovarian cancer cells were engrafted (3.5 × 10^6^ cells/animal) in the left lower flank of immunocompromised female NOD.Cg-PrkdcscidIl2rgtm1Sug/JicTac mice. On day 15, when tumors reached ~5–6 mm, animals were randomized into one of the treatment groups (5–7 animals/group) and were humanized with OvCa patient-derived autologous expanded PBMCs, 5.0 × 10^6^ cells/animal. Subsequently, virus treatments with Ad5/3-E2F-d24 or Ad5/3-E2F-d24-vIL2 virus were given (1×10^9^ vp/tumor) via intratumoral injection on days 0, 3, 6, and 9 to the animals. On day 1, treatment groups received a single OvCa patient-derived TILs via intraperitoneal injection, 8.5 × 10^6^ cells/animal. A mock control group was included in the experiment and animals were humanized with pbmcs only similarly to the other groups. TILs monotherapy and mock groups were further injected intratumorally with PBS to match the tumor disruption promoted by the local virus treatments. Tumor development was assessed every two days with a digital caliper until day 12, when all animals were dispatched and tumors, blood, and selected organs were collected. **A** Schematic of the animal OvCa PDX model experiment layout. Normalized tumor volume represented as individual curves over experiment time across the experimental groups: **B** mock, **C** autologous TILs monotherapy, **D** TILs plus Ad5/3-E2F-d24 virus, and **E** TILs plus Ad5/3-E2F-d24-vIL2 virus. **F** Combined tumor progression in response to therapies. Biodistribution analyses by flow cytometry of (**G**) CD4+ T cells, (**H**) CD8+ T cells, and (**I**) CD56+ cells percentage levels in mice´s blood and tumors. Combined tumor growth statistical significance was analysed by two-way ANOVA and bar graphs by unpaired *T* test with Welch’s correction and presented as mean +− SEM. *p < 0.05, **p < 0.01, ***p < 0.001, ****p < 0.0001.

To confirm that the therapeutic benefits of adding vIL-2 virus to TILs adoptive cell therapy could be extended to an in vivo setting, we performed an animal experiment using a PDX OvCa model in mice. After tumor establishment with an OvCa patient-derived cell line, immunocompromised female mice were humanized with a single injection via i.p. using the same patient´s expanded PBMCs, one day before the treatments started. On day 0, mice received the first virus i.t. injection, followed by another three i.t. virus injections given every three days. On day 1, a single injection of expanded patient´s autologous TILs were administrated to the animals via i.p. and tumor development was followed every two days until experiment termination on day 12 (Fig. 4A). Individual tumor growth curve results confirmed the absence of tumor control in most of mock treated animals (Fig. 4B). Similar results were observed in animals treated only with TILs adoptive cell therapy (Fig. 4C). The addition of backbone virus to the TIL therapy improved the OvCa tumor control, however, tumor relapse was noted in one of the treated animals (Fig. 4D). Best tumor control was achieved in the group treated with the vIL-2 virus plus TILs, as all animals responded to the treatment until the experiment conclusion (Fig. 4E). Combined tumor growth analysis shows improved response with statistical significance (*p* < 0.05) in the virus backbone plus TILs therapy group in comparison to mock control group and TILs monotherapy (Fig. 4F). Of note, improved overall tumor response was also obtained when vIL-2 virus was added to TIL therapy, with statistical significance being noted in comparison to mock group and TILs monotherapy treatment (*p* < 0.01).

Results on lymphocyte biodistribution show that frequencies of CD4+ T and CD8+ T cells are significantly higher (*p* < 0.05) in blood than in tumors in mock, TILs monotherapy, and TILs plus backbone treated groups (Fig. 4G, H). Interestingly, treatment with vIL-2 virus plus TILs promoted equally high proportions of CD4+ T and CD8+ T cells in blood and in tumors, although with no statistical difference observed. Moreover, these cells were augmented also when compared to the other experimental groups, especially when compared to mock and TILs monotherapy. No differences were observed in the biodistribution of CD56+ cell in blood and tumors, except in group treated with TILs plus virus backbone, where higher percentage of (*p* < 0.001) CD56+ was observed in blood than in tumors (Fig. 4I). In addition, no toxicity associated to the treatments were observed in the animals.

### Ad5/3-E2F-d24-vIL2 virus treatment stimulates high granzyme B production by CD4+ T, CD8+ T, and NK cells in ovarian cancer tumors treated with TILs therapy

**Fig. 5:**
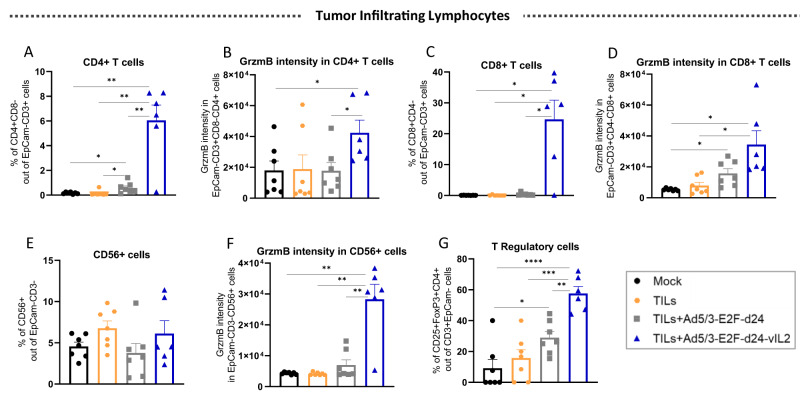
Overview on the tumor infiltrating lymphocytes status after combination therapy treatment in vivo. Tumors were collected on day 12, processed into single-cell suspensions, and stained for flow cytometry analyses. **A** Percentage of CD4+ T cells in tumors. **B** Granzyme B MFI in CD4+ T cells. **C** Percentage of CD8+ T cells. **D** Granzyme B intensity in CD8+ T cells. **E** Percentage of CD56+ cells. **F** Granzyme B intensity in CD56+ cells. **G** Levels of T Regulatory cells infiltrating OvCa PDX tumors. Data sets were analysed for statistical significance by unpaired *T* test with Welch’s correction and presented as mean +− SEM. *p < 0.05, **p < 0.01, ***p < 0.001, ****p < 0.0001.

In order to understand the immune mechanisms underlying the anti-tumor response generated by the combination therapy in vivo, we further studied TILs harvested on day 12. Intratumoral frequency of CD4+ T cells were found to be the highest in the tumors treated with TILs and vIL-2 virus (*p* < 0.01) (Fig. 5A). To a lower extent, TILs plus virus backbone also had higher proportion of CD4+ cells than the TILs monotherapy and mock groups (*p* < 0.05). Cytotoxic CD4+ T cells were identified in higher proportions in the treated tumors when vIL-2 virus was added to the TIL therapy than in the other therapies (*p* < 0.05) (Fig. 5B). A statistically significant (*p* < 0.05) increased presence of tumor-infiltrating CD8+ T cells was only detected in vIL-2 virus plus TILs treated animals (Fig. 5C). Similarly, GrzmB intensity in CD8+ T cells was identified in higher frequency when the vIL-2 virus in combination with TIL therapy was used relative to the other groups, and with statistical significance (*p* < 0.05) when compared to mock and TILs monotherapy groups (Fig. 5D). Curiously, no difference was observed in the proportion CD56+ cells infiltrating the tumors irrespective of the treatment used (Fig. 5E). However, when vIL-2 virus was used in conjunction with TIL therapy, a statistically significant (*p* < 0.01) increase in GrzmB intensity of CD56+ cells was detected in treated tumors when in comparison to the other treatment groups and mock (Fig. 5F). Lastly, a higher proportion of TReg cells infiltrating tumors was found in the vIL-2 virus plus TILs group, with statistical significance (*p* < 0.01) observed when compared to the other experimental groups (Fig. 5G). Similarly, TILs therapy plus virus backbone treated tumors had statistically significant higher percentages of TReg cells than in mock control group (*p* < 0.05).

## Discussion

Given the therapeutic impact that adoptive TILs transfer has had in the treatment of melanoma with cases of durable responses reported, and the demonstration of tumor-reactive lymphocytes infiltrating OvCa tumors, adoptive TILs transfer has been studied as an immunotherapeutic option for OvCa treatment [14, 36, 37]. In spite of improvements in protocols for TILs production and enrichment for ovarian tumor-specific T cells, the clinical results have been suboptimal [6, 8, 11, 38]. The limited ability of infused TILs to overcome the OvCa TME immunosuppression mediated by TReg cells, MDSCs, and TAMs, help to explain the current clinical findings [36].

The present study proposes the use of an oncolytic adenovirus encoding a human vIL-2 cytokine as a combination strategy to bolster TILs adoptive cell therapy anti-tumor response for the treatment of OvCa malignancies. In this regard, the genetically modified adenovirus backbone, Ad5/3-E2F-d24, used here represents a therapeutic asset to reshape the OvCa’s hostile TME [33]. The virus backbone selectively replicates in cancer cells and promotes enhanced infectivity in OvCa cells, concomitantly to the innate adenovirus ability to lyse cancer cells upon infection. In fact, adenovirus-induced cell killing is associated with shedding of immunogenic molecules involved in danger signaling cascades and tumor epitope spreading, which in turn stimulates the host immune system engagement and de novo anti-tumor generation [25, 26, 39].

Interestingly, our results are in line with these notions, as when the virus backbone treatment is accompanied by TIL therapy, enhancement of cell killing is observed ex vivo and better tumor control achieved in vivo. However, consistent anti-tumor killing and lymphocyte cytotoxicity engagement is observed only when the virus construct encodes a vIL-2 cytokine, as shown in OvCa co-cultures and in the OvCa PDX in vivo tumor model. Curiously, the vIL-2 virus or backbone virus monotherapies do not seem to deliver the same efficacy when compared to their TIL combination counterparts, in nearly half of the patient samples studied. The rapid cancer cell proliferation that is often observed in OvCa tumors, especially in more advanced stages of the disease, such as in metastatic lesions, can be a contributing factor for limiting the viruses monotherapy benefits [20]. Of note, in the clinic this would not represent a therapeutic impediment considering that oncolytic virus treatments are administered multiple times [40–42]. In the TILs only group, partial cell killing was observed in most of the studied OvCa co-cultures and no in vivo tumor control was shown. Yet, it is important to stress that the limited number of samples included in the present study limits the extrapolation of observed results with expected patient outcome in the clinic when treated with the proposed combination.

From an immunotherapeutic perspective, the proposed combination therapy was the only therapy able to engage consistent cytotoxicity response in NK cell, CD4+ T, and CD8+ T cell lymphocyte subsets in the tested assays. Notably, these promising findings initially observed in treated OvCa tumor digests were confirmed by the in vivo animal experiment results. In fact, the enhanced cytotoxicity of said effector lymphocytes was associated with improved anti-tumor response in both ex vivo and in vivo experimental contexts. Of note, we did not observe any metastasis formation in any of the experimental animals enrolled in the PDX in vivo study. Altogether, these results support the use of the vIL-2 virus to enhance TIL therapy cytotoxicity for the treatment of human OvCa tumors. Future studies including caspase-3 marker can elucidate the mechanisms of cell death triggered by the virus when combined with TIL therapy.

In addition to high proportions of CD4+ T and CD8+ T cells infiltrating PDX tumors in vivo, the percentage of TReg cells was also increased by the proposed vIL-2 plus TILs combination treatment compared to the other experimental groups. Such results are expected in view of the marked participation of CD4+ T and CD8+ T cells in the anti-tumor response and their role as important sources of wild-type IL-2 (wtIL-2) cytokine production upon cell activation [31, 43]. Once secreted in the TME, wtIL-2 becomes readily available for consumption by any infiltrating lymphocytes expressing an IL-2R, including the TReg cells [44]. In the present work, the in vivo study of intratumoral immune cells was limited to the lymphocytes injected into the animals. However, the combination therapy effects in MDSCs and other immune cells infiltrating the OvCa TME could be explored in future syngeneic studies.

It is important to highlight that our vIL-2 virus technology neither prevents production of wtIL-2 cytokine by the host immune cells nor inhibits wtIL-2 binding to its cell receptor. In fact, our vIL-2 virus aims to express continuously the vIL-2 cytokine in the TME to be consumed by effector lymphocytes only. Supporting this idea, we previously demonstrated that the relative expression of vIL-2 cytokine transgene and the host wtIL-2 gene expression were equally high in hamster tumors treated with vIL-2 virus, which was sufficient to trigger better tumor control and survival compared to its wtIL-2 virus counterpart [28]. Moreover, the therapy with vIL-2 demonstrated to be safe in the treated animals as monotherapy, and here we did not observe any systemic adverse reactions due to the treatment or at the local site of the virus injections Here, the potent pro-inflammatory response mainly mediated by the increased cytotoxicity of NK cell, CD4+ T and CD8+ T cells have been able to outperform the rapid tumor growth and TReg cell immunosuppression in the combination treated animals.

The presence of intratumoral lymphocytes in OvCa tumors has prognostic value for increased survival and it is a natural requirement for TIL cell therapy [45]. In the present study, all OvCa samples had detectable TILs at the baseline with counts for CD4+ T, CD8+ T and NK cells varying greatly across all samples, with NK cells being found in lower frequency than said T cells, and even absent in 4 out of 11 samples in the IHC slides studied. Moreover, 10 out of 11 samples presented high ratio of CD4+/CD8+ T cells, confirming the usual profile of CD4+ T lymphocytes infiltrating OvCa tumors [21, 36, 38]. Interestingly, the initial TIL infiltration did not seem to affect treatment outcome when the OvCa tumor digests were treated with vIL-2 virus in combination with TILs therapy.

Another important aspect to consider is the initial patient diagnosis, neo-adjuvant chemotherapy, and disease stage of OvCa samples included here. Majority of the samples were collected from metastatic lesions, with most (10 out 12) being already at stage IVB of the disease. Despite the initial prognosis, nearly all those specimens showed better OvCa cell killing when the vIL-2 virus was added to the TIL therapy, compared to the other treatment groups. In fact, these are promising results with clinical importance in view of limited current curative options and poor prognostic survival for patients with advanced OvCa [10]. Future studies evaluating the impact of the neo-adjuvant chemotherapy to the combination of vIL-2 virus plus TILs anti-tumor response are needed.

Adoptive cell transfer (ACT) with TILs has demonstrated reproducible clinical results in certain solid tumors and in multiple myeloma, however, it has not yet achieved major clinical benefits in OvCa. Since the publication of the first trial results in the early 1990s [9], many approaches have been explored in order to reduce the TILs expansion time [8, 12] and to optimize trial protocols with the use of adjuvant and/or lymphodepleting chemotherapy [11, 46], exogenous administration of IL-2 cytokine [11], or even co-treatment with chemotherapy in addition to immune checkpoint inhibitors [37]. Unfortunately, the overall results have mainly demonstrated short-time stable disease and sporadic complete responses [9, 11, 37]. Taking into account that adoptive TIL therapy is an autologous immunotherapy, and essentially a personalized therapy, some of these variations (patients unique TME, tumor mutational load, TILs cell yield, and tumor-specific TILs) are inherent to each patient and difficult to modify if no further cell engineering is employed [47].

Here, we propose a different approach that aims to improve the therapeutic benefits of TILs in advanced stage human OvCa tumors. Ad5/3-E2F-d24-vIL2 showed consistent improvement of TIL cytotoxicity through GrzmB production detected in NK, CD4+ T and CD8+ T cells in treated advanced stage OvCa tumors specimens. Likewise, the therapeutic benefit of combining the vIL-2 virus with TILs was confirmed in a PDX OvCa tumor model, resulting in improved tumor control and enhanced TIL cytotoxicity, compared to TIL monotherapy.

In conclusion, the results presented here demonstrate the clinical potential of Ad5/3-E2F-d24-vIL2 to be used in combination with TIL cell therapy for the treatment of OvCa patients that currently lack therapeutic options. However, the number and volume of OvCa patient samples available constituted a limiting factor for the immune studies that could be done on OvCa patient specimens. This in turn influences data interpretation and generalizability. Therefore, future studies should include more OvCa specimens, optimally with higher tumor volume, when studying Ad5/3-E2F-d24-vIL2 virus in combination with TIL therapy for the treatment of advanced OvCa cancer.

### Supplementary information

Supplements-CGT

## Data Availability

All data generated or analysed during this study are included in this published article [and its supplementary information files].
