# Supplementary material for: Improving the cytotoxic response of tumor-infiltrating lymphocytes towards advanced stage ovarian cancer with an oncolytic adenovirus expressing a human vIL-2 cytokine
Source: Cancer Gene Ther. 2023 Sep 4;30(11):1543–53. doi: 10.1038/s41417-023-00658-3 (PMC10645590; doi:10.1038/s41417-023-00658-3)
Supplement: Supplementary file 1 — Supplements-CGT [file 41417_2023_658_MOESM1_ESM.pdf]

Improving the cytotoxic response of tumor-infiltrating lymphocytes towards advanced stage ovarian cancer with an oncolytic adenovirus expressing a human vIL-2 cytokine

Quixabeira DCA[1,2], Jirovec E[1], Pakola S[1], Havunen R[1,2], Basnet S[1], Santos JM[1,2], Kudling TV[1], Clubb JHA[1,2], Haybout L[1], Arias V[1], Grönberg-Vähä-Koskela S[1, 3], Cervera-Carrascon V[1,2], Pasanen A[4], Anttila M[5], Tapper J[6], Kanerva A[6], and Hemminki A[1,2,3*]

Supplements

Supplementary Table 1- Flow cytometry panel				
Antigen	Flurochrome	Clone	Catalogue Number	Provider
CD3	AF700	SK7	344822	Biolegend
CD4	BV570	RPA-T4	300534	Biolegend
CD8	BV510	RPA-T8	563256	BD
PD-1	APC-Cy7	EH12.2H7	329922	Biolegend
CD25	BV711	M-A251	356138	Biolegend
FoxP3	PE-Dazzle	206D	320126	Biolegend
GrzmB	PE	GB11	561142	BD
EpCam	PE-Dazzle	9C4	324232	Biolegend
CD56	BV510	HCD56	318340	Biolegend

Supplementary Table 1. List of Antibodies and fluorochromes used for the immune cells studies.

Supplementary Table 2- IHC antibody panel		
Antibody	Dilution used	Provider
CD4 (104R-16)	1:100	Cell Marque
CD8 (NCL-CD8-4B11)	1:50	N-C
CD56 (156R-96)	1:500	Cell Marque
PD-L1 (741-4860)	Ready to use	Roche

Supplementary Table 2. List of antibodies used for the immunohistochemistry staining of OvCa slides.
